# Exploring distinct properties of endometrial stem cells through advanced single-cell analysis platforms

**DOI:** 10.1186/s13287-023-03616-w

**Published:** 2023-12-20

**Authors:** Jin Woo Lee, Hwa-Yong Lee

**Affiliations:** 1 Department of Health Sciences and Technology, GAIHST, Gachon University, Incheon, 21999 Republic of Korea; 2 Department of Molecular Medicine, School of Medicine, Gachon University, Incheon, 406-840 Republic of Korea; 3 Division of Science Education, Kangwon National University, Chuncheon, 24341 Republic of Korea

**Keywords:** Endometrial stem cells, Single-cell analysis, ScRNA-seq, ScATAC-seq, Spatial transcriptomics

## Abstract

The endometrium is a dynamic tissue that undergoes cyclic changes in response to ovarian hormones during the menstrual cycle. These changes are crucial for pregnancy establishment and maintenance. Endometrial stem cells play a pivotal role in endometrial regeneration and repair by differentiating into various cell types within the endometrium. However, their involvement in endometrial disorders such as endometriosis, infertility, and endometrial cancer is still not fully understood yet. Traditional bulk sequencing methods have limitations in capturing heterogeneity and complexity of endometrial stem cell populations. To overcome these limitations, recent single-cell analysis techniques, including single-cell RNA sequencing (scRNA-Seq), single-cell ATAC sequencing (scATAC-Seq), and spatial transcriptomics, have emerged as valuable tools for studying endometrial stem cells. In this review, although there are still many technical limitations that require improvement, we will summarize the current state-of-the-art single-cell analysis techniques for endometrial stem cells and explore their relevance to related diseases. We will discuss studies utilizing various single-cell analysis platforms to identify and characterize distinct endometrial stem cell populations and investigate their dynamic changes in gene expression and epigenetic patterns during menstrual cycle and differentiation processes. These techniques enable the identification of rare cell populations, capture heterogeneity of cell populations within the endometrium, and provide potential targets for more effective therapies.

## Introduction

The endometrium is a dynamic tissue that undergoes cyclic changes in response to ovarian hormones during the menstrual cycle. These changes are critical for the establishment and maintenance of pregnancy [1]. Endometrial stem cells are known to play a key role in endometrial regeneration and repair [2]. It has been shown that endometrial stem cells contribute to this process by differentiating into endometrial stromal cells [3], glandular epithelial cells [4], and vascular smooth muscle cells [5]. These differentiated cells then form a new functional layer of the endometrium. These cells have also been implicated in the pathogenesis of endometrial disorders such as endometriosis [2] and endometrial cancer [6]. However, traditional bulk sequencing methods, which analyze the average gene expression or epigenetic pattern across a population of cells, are limited in their ability to capture the heterogeneity and complexity of endometrial stem cell populations within the dynamic endometrial tissue during menstrual cycle and differentiation process [7, 8].

In this context, various single-cell analysis techniques such as single-cell RNA sequencing (scRNA-Seq) [9, 10], single-cell ATAC sequencing (scATAC-seq) [10], and spatial transcriptomics [11] have been recently developed and become increasingly important for studying endometrial stem cells. Although there are still many technical limitations that require improvement, the utilization of single-cell analysis allows for addressing cellular heterogeneity by grouping similar cells together while separating dissimilar cells. The advantages and disadvantages of the single-cell analysis platform are summarized in Table 1 for a comprehensive overview. This approach enables computational construction of a pseudotime trajectory for a biological process based on transcriptomic capture of unsynchronized cells from the tissue. Further analysis can be performed to resolve functional processes through evaluation of differential gene expression across identified clusters or trajectories [9]. Therefore, single-cell analysis platforms enable us to identify rare cell populations and capture heterogeneity of cell populations within the endometrium, which might be targeted for more effective therapies for endometrial diseases [12]. For example, scRNA-Seq has been used to identify distinct subpopulations of endometrial stromal cells including proliferative and secretory stromal cells during menstrual cycle and to characterize gene expression changes that occur during endometrial differentiation [13, 14]. The inclusion of epigenetic data such as chromatin accessibility is crucial for providing a comprehensive understanding of the regulatory landscape during development. In recent studies, researchers have expanded transcriptomic analyses to incorporate single-cell assay for transposase-accessible chromatin sequencing (scATAC-Seq). This powerful method allows for characterization of potential gene regulatory networks through identification of changes in accessible chromatin that occur with cell-state changes [15]. By combining scATAC-Seq and scRNA-Seq, an unparalleled resolution can be achieved for a developing human endometrium. Indeed, scATAC-seq has been used to identify cell type-specific epigenetic changes during menstrual cycle and to identify putative regulatory elements associated with endometrial differentiation [12, 16].

**Table 1:** The advantages and disadvantages of the single-cell analysis platform

Advantages of single-cell analysis	Disadvantages of single-cell analysis
High Resolution: Provides detailed insights into individual cell characteristics	Technical Challenges: Complex and resource-intensive procedures may pose technical difficulties
Heterogeneity Exploration: Allows for the study of cellular diversity within tissues	Cost: Can be expensive, particularly when analyzing large numbers of single cells
Precision Medicine: Facilitates personalized treatment strategies based on individual cell profiles	Data Analysis Complexity: Handling and interpreting large datasets require advanced bioinformatics expertise
Rare Cell Detection: Enables the identification of rare cell populations within a sample	Cell Isolation Issues: Challenges in isolating and capturing individual cells without bias
Early Disease Detection: May detect cellular changes at early stages of disease development	Limited Sample Size: Some techniques may require a significant number of cells, limiting applications in samples with low cell numbers
Cell Fate Mapping: Helps in understanding cellular developmental trajectories	Cell Stress Response: The process of isolating single cells can induce stress-related changes in gene expression
Identification of Cell Types: Allows for the identification and characterization of specific cell types	Standardization Challenges: Lack of standardized protocols may lead to variability in results between studies

In this review, we will summarize current state-of-the-art techniques for various single-cell analyses of endometrial stem cells and their related diseases. We will discuss recent studies that have used single-cell analysis to identify and characterize distinct endometrial stem cell populations and to investigate dynamic changes in gene expression and epigenetic patterns of endometrial stem cells during menstrual cycle and differentiation into specific cell lineages. We will also highlight potential roles of endometrial stem cells in the development and progression of various uterine diseases through their ability to accumulate genetic mutations, express genes associated with uterine diseases, and interact with other cells within tissue microenvironment. Overall, this review demonstrates the power of various single-cell analyses for advancing our understanding of the molecular mechanisms underlying endometrial development and function as well as for developing new therapies for endometrial stem cell-related disorders.

## Why single-cell analysis can be an effective strategy for studying endometrial stem cells?

Single-cell analysis has emerged as a powerful tool for investigating properties of endometrial stem cells. Single-cell analysis provides their gene expression, epigenetic modifications, protein expression, and other molecular features. One advantage of single-cell analysis is its ability to identify and characterize rare or heterogeneous cell populations that might be missed by bulk tissue analysis [7]. For example, endometrial stem cells are a relatively rare population within the endometrium. It can be difficult to isolate and study them using traditional methods. Single-cell analysis allows researchers to identify and study these cells with greater precision. Another advantage of single-cell analysis is its ability to reveal molecular mechanisms underlying stem cell differentiation and self-renewal [17]. By comparing gene expression profiles of stem cells at different stages of differentiation, researchers can gain insights into regulatory networks and signaling pathways involved in these processes. Several reasons outlining the usefulness of single-cell analysis in endometrial stem cell research will be discussed.

### Various types of endometrial stem cells

Endometrial stem cells constitute a subset of cells endowed with the ability for self-renewal and multilineage differentiation into various types of cells constituting the endometrial tissue [18, 19]. In fact, endometrium encompasses several types of tissue-resident stem cells, such as epithelial-like stem cells [4], stromal-like stem cells [3], and perivascular endometrial stem cells [20]. Each of these stem cell types possesses distinctive molecular and functional properties.

Stem cells with epithelial characteristics are believed to reside within the basal layer of the endometrial tissue, situated proximately to the basal laminae. These epithelial-like stem cells exhibit the capacity for both self-renewal and multilineage differentiation, enabling them to generate the glandular epithelium of the endometrial tissue. Epithelial-like stem cells undergo regulation through an intricate interaction with various endocrine and paracrine factors, such as hormones and/or growth factors from the adjacent immune and stromal cells. For instance, Janzen et al. revealed that the self-renewal capacity and differentiation potential of EpCAM/CD44 positive epithelial-like stem cells, along with the Wnt/β-catenin signaling and its downstream regulators such as Axin2, c-Myc, CD44, and ID2, can be regulated by progesterone and estrogen [21].

Stem cells with stromal characteristics within endometrial tissue are believed to reside in the perivascular region of the stromal area, which functions as connective tissue providing structural support for endometrial blood vessels and glands [22]. These stem cells possess the capability to differentiate into various cell types such as endothelial cells, fibroblasts, and smooth muscle cells [23]. Moreover, stromal-like stem cells exhibit immunomodulatory characteristics, potentially playing a significant role in the regulation of immune responses within the endometrial tissue. According to the results of Leñero et al., therapeutic potential of CD146^+^ stromal-like stem cells were found to be predominantly facilitated by secretion of a blend of enriched factors, including let-7e-5p, miR-182-3p, miR-320e, and miR-378 g [24]. These secretory factors specifically interact with the immune system and influence angiogenesis by modulating macrophage polarization, T cell activity, and transcriptional regulation of various immune regulatory cytokines including IL-1β, IL-6, and TNF-α.

Perivascular stem cells are situated within the perivascular region of the endometrial tissue, displaying the capacity to differentiate into both stromal and epithelial cell types [25, 26]. It is believed that these stem cells contribute to the establishment and upkeep of the vasculature within the endometrium. These stem cells are identified by the expression of distinct cell surface biomarkers, including CD146, PDGFRβ, and SUSD2 [2]. Li et al. employed flow cytometry, utilizing antibodies against CD10, CD13, CD44, CD73, CD90, and CD105, to isolate perivascular endometrial stem cells in humans. They illustrated the cells' ability to undergo differentiation into adipocytes, neuron-like cells, and osteoblasts [27].

### High heterogeneity of endometrial tissue

The human endometrium exhibits remarkable cellular diversity, with various cell types contributing to its complex functions. Epithelial cells lining the luminal surface undergo cyclic changes and participate in embryo implantation and glandular secretion [28]. Stromal cells that are highly plastic in nature can support tissue remodeling and respond to hormonal cues [29]. Immune cells including macrophages, natural killer cells, and T cells can interact with other cell types to regulate immune responses and tissue remodeling [30]. Endothelial cells ensure proper vascularization and nutrient exchange. Supporting cells such as smooth muscle cells, perivascular cells, and fibroblasts provide structural support [31]. Understanding the dynamic interplay and functional roles of these diverse cell populations within the endometrium is crucial for comprehending endometrial biology, reproductive processes, and associated disorders. Currently, understanding the generation of cellular diversity from a single stem cell, regulatory mechanisms governing dynamic tissue regeneration, and utilization of this diversity to mount appropriate responses to external perturbations are central challenges in endometrial research [32].

**Fig. 1:**
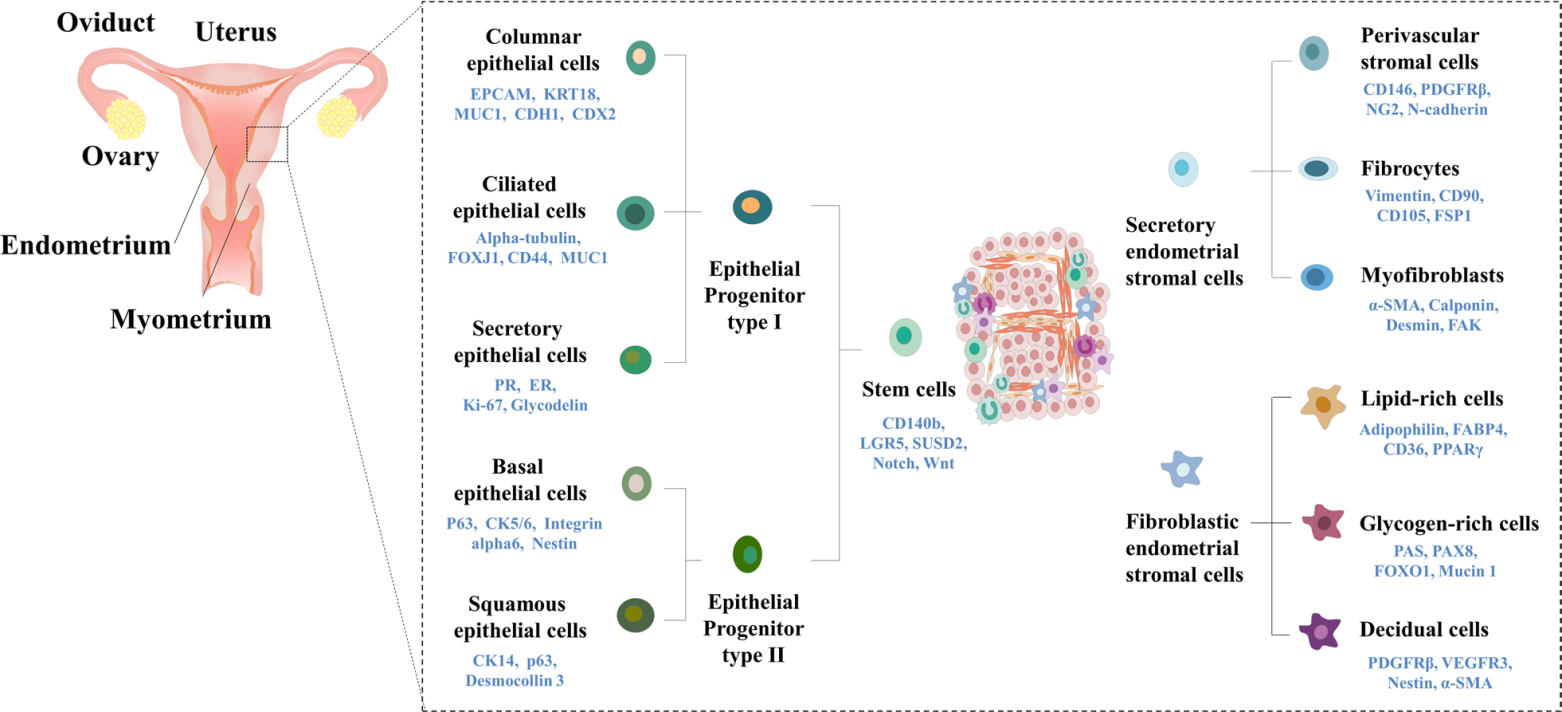
Comprehensive landscape of identified cell types in normal endometrial tissue and the current understanding of their lineage hierarchy. This figure provides an overarching depiction of the diverse cell types recognized within normal endometrial tissue, along with the existing perspective on their lineage relationships. The pivotal role of endometrial stem cells as precursors for both epithelial and stromal cell lineages is outlined, underscoring their significance in the generation of various differentiated cell types, each characterized by distinctive surface markers. Specifically, Type I epithelial progenitor cells exhibit the capacity to undergo differentiation into columnar epithelial cells, ciliated epithelial cells, and secretory epithelial cells. Meanwhile, Type II epithelial progenitor cells demonstrate the potential to differentiate into basal epithelial cells and squamous epithelial cells. Furthermore, the lineage progression extends to secretory endometrial stromal cells, which exhibit the capability to differentiate into perivascular stromal cells, fibrocytes, and myofibroblasts. Additionally, fibroblastic endometrial stromal cells manifest the ability to undergo differentiation into distinct cell types, including lipid-rich cells, glycogen-rich cells, and decidual cells. The figures presented in this article were crafted by our group

Cellular diversity within the endometrium plays a crucial role in maintaining tissue functionality and adapting to environmental stimuli. However, unraveling mechanisms responsible for generating and regulating this diversity remains a significant scientific endeavor. To address these issues, Díaz-Gimeno et al. have performed comprehensive transcriptomic profiling of the endometrium during various time points encompassing the mid-secretory phase. Their research unraveled certain levels of molecular characteristics of the endometrium associated with the crucial window of implantation (WOI), a narrow timeframe during which the endometrium is receptive to embryo implantation [33]. However, simple comparative analysis of the transcriptome with traditional approaches between patients and healthy controls has provided limited insights into the underlying molecular mechanisms involved in uterine disorders such as endometriosis, recurrent implantation failure (RIF), and recurrent pregnancy loss (RPL) [34]. Cells within a given tissue have traditionally been perceived as functionally identical units. Conventional detection methods often rely on capturing overall response of these cells [35]. Thus, conventional technologies commonly employ bulk population-level measurements, neglecting distinctive cellular behaviors arising from cell-to-cell variations such as cell metabolism, differentiation, and growth [36]. The collective functionality of a complex tissue is actually derived from a heterogeneous population of cells that exhibit subtle variations among them. Moreover, the endometrium undergoes dynamic changes during the menstrual cycle, which further adds to its complexity (Fig. 1).

**Fig. 2:**
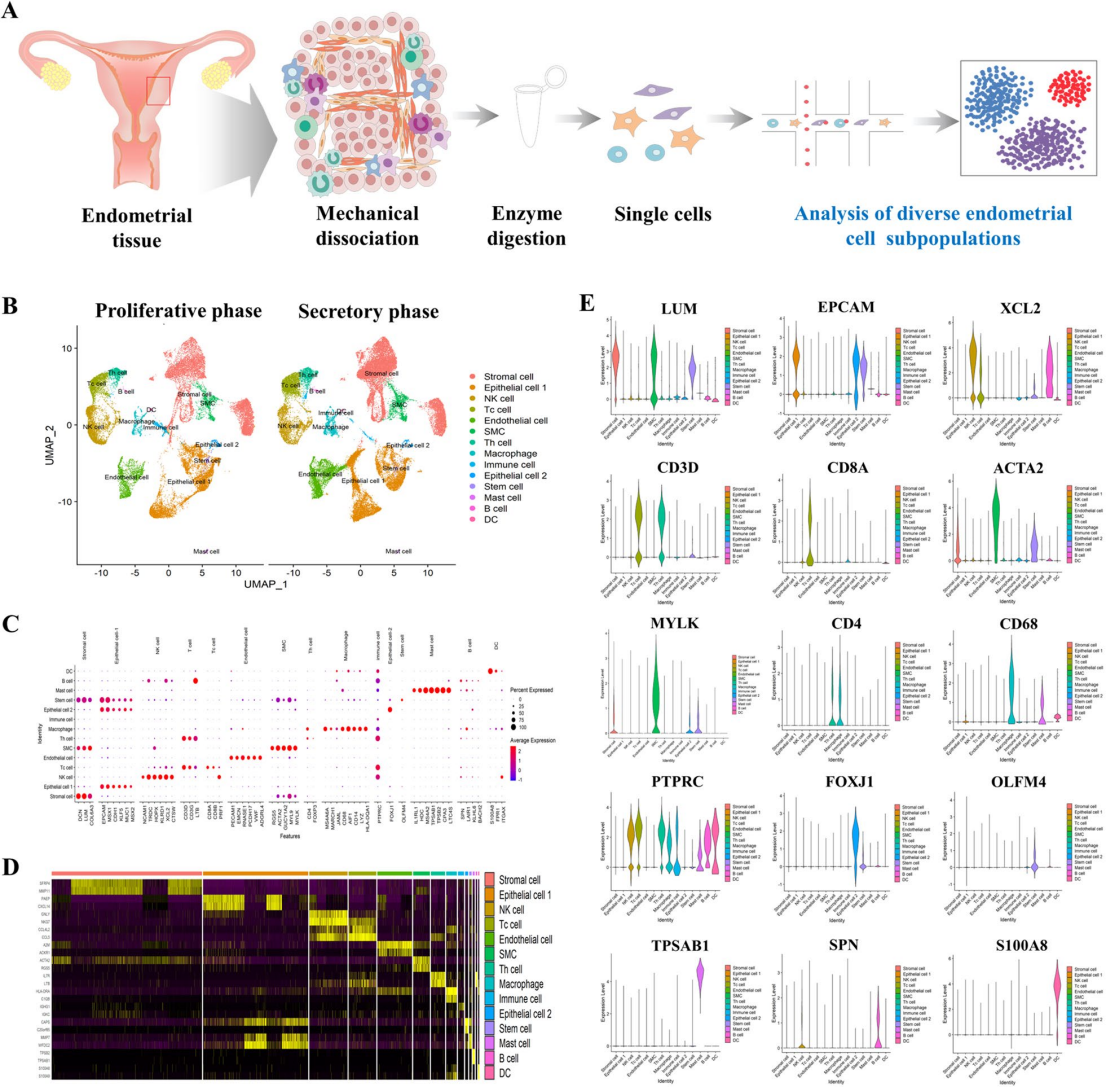
Profiling diverse endometrial cell types at single-cell resolution throughout the menstrual cycle. Schematic outlining the sequential steps involved in the isolation and individual-level analysis of various cell populations from healthy endometrial tissue (**A**). UMAP visualization depicting distinct cell clusters identified in endometrial samples collected during both the proliferative and secretory phases of the menstrual cycle. A total of 14 distinct endometrial cell clusters representing different menstrual cycle stages are highlighted on the UMAP plot (**B**). Distinctive expression patterns of multiple biomarkers for each cell type were explored. Each data point on the visualization corresponds to an individual gene, with the intensity of color reflecting the average expression level. Additionally, the size of each data point corresponds to the proportion of cells within the cell type that exhibit expression of the given gene (**C**). Heatmap of top differentially expressed genes in each endometrial consistent cell types during both the proliferative and secretory phases (**D**). Visualization of expression patterns for differentially expressed genes (DEGs) among different endometrial cell types during both the proliferative and secretory phases, presented through violin plots (**E**). The figures presented in this article were crafted by our group

In this context, single-cell analysis provides a powerful approach for uncovering the hidden diversity and complexity within tissues. By studying individual cells instead of averaging their characteristics in the tissue, researchers can discern heterogeneity and understand functional implications of these individual cellular differences. The ability to study transcription patterns and epigenetic regulation in single cells was limited by technological constraints in the past. However, recent advances in bioanalytical technologies have enabled the study of transcription pattern [37] and epigenetic regulation [38] in single cells, which in turn allow researchers to profile gene expression and signaling pathways of individual cells in a heterogeneous population, thereby providing a more detailed understanding of the heterogeneity of the endometrium at a single-cell level. By examining gene expression profiles of individual cells, researchers can identify cell subpopulations and their molecular characteristics, including their differentiation potential, proliferation rates, and interaction with neighboring cells. For example, Garcia-Alonso et al. have integrated scRNA-Seq and spatial transcriptomics to investigate the cellular composition and heterogeneity within the endometrium. They analyzed approximately 100,000 cells from proliferative and secretory phases of the endometrium sourced from 15 women in their reproductive age [11]. Through a meticulous examination of these cells, they successfully identified and characterized 14 distinct clusters belonging to the following five major cell type categories: (a) epithelial cells (b) endothelial cells, (c) immune cells, (d) stromal cells, and (e) supporting cells (including smooth muscle cells, perivascular cells, and fibroblasts expressing the cell marker C7) [11]. Recent advancements in single-cell technologies have facilitated more detailed investigations into cellular composition and heterogeneity within the endometrium, providing valuable insights into previously unrecognized cell subsets and their functional roles (Fig. 2).

### Identification of rare stem cell subpopulations within endometrial tissue

The human endometrium possesses a remarkable regenerative capacity, undergoing significant remodeling and regeneration during each menstrual cycle, resulting in the generation of 4–10 mm of a mucus layer [39]. The ability of the endometrium to undergo such extensive regeneration is attributed to the presence of resident epithelial progenitors and stromal-like endometrial stem cells. [2, 40]. The identification of endometrial stem cells with the capacity to generate substantial colonies is a rare occurrence, comprising only a small fraction of the overall cell population. Specifically, the presence of these stem cells has been documented at frequencies of approximately 0.08% for epithelial cells and 0.02% for stromal cells [41].

In spite of significant advancements in the study of endometrial stem cells, our understanding of their specific identity, localization, and regulatory mechanisms remains largely elusive. Conventional approaches such as bulk RNA sequencing, while valuable, often obscure the presence and distinct gene expression patterns of these infrequent cells within the complex endometrial tissue. However, unlike traditional methods, recent application of single-cell analysis enables identification and quantification of these rare stem cell subpopulations within the endometrial tissue by assessing gene expression, epigenetic modifications, and cell surface markers at the individual cell level without needing enrichment or isolation. For example, Garcia-Alonso et al. have employed scRNA-Seq to investigate epithelial cell populations within primary endometrial tissues and cells and identified potential epithelial stem cell populations and their associated markers. Notably, they revealed that a SOX9^+^ epithelial subpopulation was a potential driver of epithelial regeneration within the endometrium [11].

They discovered distinct subsets within the identified SOX9^+^ epithelial subpopulation, including noncycling SOX9^+^/LGR5^+^ cells located in the surface epithelium, SOX9^+^/LGR5^−^ cells present in the basal glands, and proliferating SOX9^+^ cells found in the regenerating superficial endometrial layer. Such cellular dynamics and heterogeneity within the SOX9^+^ subpopulation provide valuable insights into the mechanisms of epithelial regeneration and underscore the complexity of endometrial stem cell behavior in different regions of the endometrium. Queckbörner et al. have found the presence of multiple stromal cell subpopulations in addition to known endometrial cell types. These stromal subpopulations exhibited distinct characteristics, suggesting the existence of specific stromal niches with potential regulatory roles in inflammation and extracellular matrix composition [13]. They also identified ten different stromal cell clusters and two subsets of pericytes, highlighting the diversity within the stromal compartment. Furthermore, they delineated the diversity of cell clusters and established lineage trajectories utilizing various analytical platforms, including SingleR, Seurat, and Velocyto [13]. Wang et al. have also undertaken comprehensive analysis of human endometrium at single-cell level throughout the menstrual cycle. Notably, they identified transcriptomic transformations associated with critical events such as opening of the window of implantation, a pivotal phase in the endometrial preparation for embryo attachment. Additionally, they provided a systematic single-cell transcriptomic delineation of endometrial transformation, enabling a detailed understanding of diverse changes observed in various cell types, cellular states, growth patterns, and differentiation processes throughout the entirety of the human menstrual cycle [42]. Cao et al. have characterized specific endometrial cell subpopulations as stem cell entities with regenerative potential by performing single-cell expression profiling analysis. However, their results raised uncertainties about the extent to which cultured putative endometrial stem cell population could faithfully represent the in vivo population of stromal cells expressing both biomarkers PDFGRB and MCAM [9].

### Functional changes of endometrial stem cells during the development of various endometrial diseases

Currently, the presence of abnormalities and mutations in endometrial stem cells is believed to be a crucial factor in the initiation and progression of various endometrial diseases, including infertility associated with a thin endometrium, endometriosis, and endometrial cancers [43]. The achievement of a successful pregnancy hinges on the growth of an embryo within an accommodating endometrium of sufficient thickness. Clinically, a thin endometrium is acknowledged as a contributing factor to infertility, recurrent pregnancy loss, and complications in placental development [44]. Consistently, Tewary and colleagues presented their research findings, highlighting a connection between reduced clonogenic populations of endometrial cells at the baseline and the varying degrees of recurrent pregnancy loss associated with impaired endometrial growth [45].

Endometriosis is a common and non-malignant gynecological disease frequently characterized by pelvic pain and infertility. This disease is defined by the existence of tissue resembling the endometrium in locations outside the normal uterus, and these ectopic tissues undergo substantial alterations influenced by hormonal fluctuations during the menstrual cycle [46, 47]. Indeed, Moggio et al. have observed that noted endometrial stem cells derived from endometriosis tissues demonstrated markedly enhanced self-renewal capacity, migratory potential, and angiogenesis when compared to stem cells from the same individual’s normal endometrial tissue or those from healthy controls [48]. This result indicates that the aberrant behavior exhibited by endometrial stem cells could potentially play a role in the development of endometriosis. Uzan et al. have observed a notable down-regulation in the expression levels of ARID1A, PTEN, and TNFα within CD73^+^/CD90^+^/ CD105^+^ endometrial stem/progenitor cells in ectopic endometriosis tissue samples when compared to normal endometrial tissue samples [49]. This result indicates that irregular expression of particular genes may make endometrial stem cells more susceptible to the development of endometriosis.

Moreover, there is a potential involvement of endometrial stem cells in the development and progression of endometrial cancer [6], as they exhibit heightened self-renewal capacity and genetic instability in certain instances. A suggested mechanism posits that genetic mutations may accumulate in endometrial stem cells, initiating the transformation of these cells from their normal state into cancerous cells. For instance, Syed et al. have proposed a hypothesis proposing the existence of stem/progenitor cells within endometrial glands that respond to Wnt signaling pathway. They identified Axin2, a recognized Wnt reporter gene, as a biomarker indicative of epithelial-like stem/progenitor cells located in the endometrial glands. [50].

### Dynamic changes in gene expression and epigenetic modifications throughout the menstrual cycle and the differentiation process

The human endometrium is a remarkable tissue that undergoes dynamic cyclic changes characterized by gradual shedding of the surface epithelium and subsequent rapid restoration of tissue homeostasis. Apart from other tissues such as the skin, endometrium has a unique ability to efficiently repair itself without leaving any scar formation. Dynamic interactions of various endometrial cellular components within the microenvironment further add to the challenge of comprehending how different cell types are mobilized and coordinated to facilitate these dynamic cyclic changes. In addition, endometrial stem cells involved in endometrial regeneration undergo dynamic changes in gene expression and epigenetic modifications throughout the menstrual cycle and the differentiation process.

In this context, single-cell analysis is a valuable technique for investigating endometrial stem cells within the endometrium due to their dynamic changes during the menstrual cycle and high heterogeneity. For example, Kirkwood et al. have analyzed endometrial tissues of mice throughout the normal cycle by integrating scRNA-Seq and lineage tracing analysis. They identified a previously unrecognized population of PDGFRb^+^ mesenchymal stem-like cells, referred to as repair-specific fibroblasts [14]. These cells exhibited the capability to undergo a transformative process from a mesenchymal state to an epithelial state, allowing them to integrate into the re-epithelialized luminal surface of the repaired tissue. This integrated analysis revealed the existence of a unique population of wound-responsive, plastic endometrial stromal fibroblasts. Remarkably, these fibroblasts demonstrated their crucial role in rapid restoration of a fully functional luminal epithelium during the process of endometrial repair [14]. Similarly, Queckbörner et al. have investigated endometrial samples obtained from healthy fertile women during the proliferative phase of the menstrual cycle by employing single-cell analysis combined with advanced bioinformatics techniques. Although the sample size was limited to *n* = 3, results provided valuable insights into the diverse landscape of stromal subsets within the endometrium [13]. These stromal cell subtypes exhibited different surface markers, cell states, ECM compositions, and immune responses. For example, ISG15^+^ stromal subtype exhibited an expression profile indicative of interferon-regulated genes and ACTA2^+^ stromal subtype displayed a consistent state characterized by a lower capacity for differentiation compared to other cell populations in the perivascular environment [13]. In addition, Kirkwood et al. have employed a transgenic reporter mouse model along with single-cell transcriptomics to establish a comprehensive repertoire of cell-specific markers for endometrial progenitor cell populations and successfully identified three distinct subpopulations of putative endometrial mesenchymal progenitors [51]. These three identified mesenchymal progenitor cell subtypes shared characteristic expression of PDGFRα and CD34 markers. However, they exhibited distinct gene expression profiles, highlighting their unique functional attributes within the endometrium. The first population demonstrated notable expression of Ngfr, Spon2, and Angptl7 genes. The second population exhibited distinct expression patterns of Cxcl14, Smoc2, and Rgs2 genes. The third population, also identified as type 1, showed specific expression of Clec3b, Col14a1, and Mmp3 genes. These genes are involved in the organization and remodeling of the extracellular matrix, suggesting the involvement of this progenitor population in maintaining structural integrity and functionality of the endometrial tissue [51].

In a previous study, Lucas et al. have observed a loss of clonal mesenchymal stem-like cells in endometrium specifically during the midluteal phase of patients with recurrent pregnancy loss [52]. However, the underlying mechanisms linking this stem cell loss to the subsequent infertility remained unclear. To address this gap, they further investigated and demonstrated that deficiency in stem/progenitor cells could contribute to a pro-senescent decidual response during the peri-implantation window by performing scRNA-Seq for decidualizing primary endometrial stromal-like stem cell cultures. This in turn led to chronic inflammation in early pregnancy, proteolysis of the decidual-placental interface, and ultimately miscarriage. They identified SCARA5 as a biomarker gene for decidual cells, providing a valuable tool for distinguishing and studying these specialized cells. On the other hand, DIO2 emerged as a marker gene for progesterone-resistant senescent decidual cells [53]. These findings contribute to our understanding of the complex interactions and molecular processes involved in decidualization, implantation, and early pregnancy.

## Various single-cell analysis methods for studying endometrial stem cells

### Single-cell RNA sequencing (scRNA-seq)

**Fig. 3:**
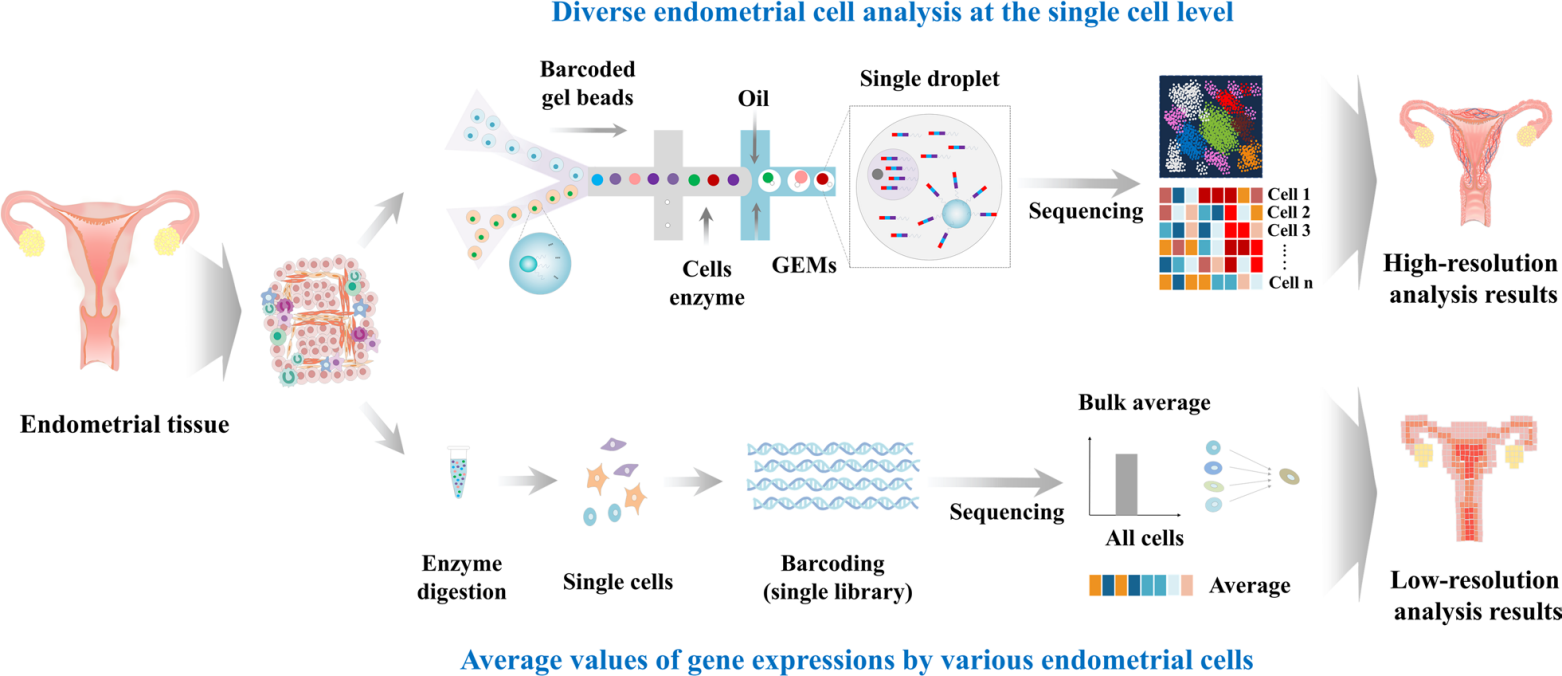
Comparison between Whole-Genome RNA Sequencing (bulk RNA-Seq) and Single-Cell RNA Sequencing (scRNA-seq) strategies. The top panel of the schematic illustrates the concept of bulk RNA-Seq, which involves sequencing the entire transcriptome of a mixed population of cells. This method provides an average measure of gene expression across all cells within the sample. In contrast, the bottom panel demonstrates the methodology of scRNA-seq, which enables a more precise exploration of cellular heterogeneity within a tissue or a specific cell subset. By individually sequencing the RNA of single cells, scRNA-seq uncovers the distinct gene expression patterns inherent to each cell. This approach, therefore, mitigates the bias introduced by bulk RNA Seq and empowers high-throughput molecular investigations with unprecedented single-cell resolution. The figures presented in this article were crafted by our group

In the last two decades, extensive research has revealed that numerous coding genes undergo dynamic changes in their expression levels within the endometrium during different phases of the endometrial cycle [42, 54, 55]. While achieving reproducible data across studies has been challenging due to technical constraints, limitations in sample availability, high heterogenicity, and dynamic change during menstrual cycle, our understanding of transcriptional networks governing functional changes in the endometrium has significantly advanced. Notably, a recent study has employed single-cell transcriptomic analysis to provide crucial insights into transcriptional profiles of individual cell types constituting the endometrium. By capturing and analyzing gene expression profiles at the single-cell level, researchers have uncovered intricacies of transcriptional dynamics within the endometrium [56]. scRNA-Seq has emerged as a powerful and transformative tool that enables comprehensive evaluation of gene expression profiles and unravels intricate cellular compositions of the endometrium to comprehend its molecular complexity in thousands of individual cells [57]. By capturing transcriptomes of individual cells, researchers have gained insights into unique molecular signatures that define different cell types, states, and subpopulations [58]. This technique has proven instrumental in characterizing heterogeneity of the endometrium as it enables identification and characterization of previously unrecognized subpopulations that might play critical roles in endometrial physiology and pathology [59] (Fig. 3).

For example, Wang et al. have characterized transcriptomic changes occurring in the functionalis layer of the endometrium, which undergoes cyclic shedding and regeneration throughout the menstrual cycle. They focused on dynamic gene expression alterations at the single-cell level in stromal and epithelial cell components [42]. They observed notable enhanced expression of PAEP, GPX3, and CXCL14 in epithelial cells. These genes serve as potential biomarkers for regulating the receptive state of the endometrium during the window of implantation. On the other hand, gene expression changes observed in stromal cells were more gradual and continuous, with genes such as FOXO1 and IL15 showing notable upregulation. Interestingly, these alterations were already detected earlier in the menstrual cycle, suggesting their involvement in preparing the endometrium for embryo implantation even before the receptive window. These transcriptomic markers could serve as valuable tools for diagnosing impaired endometrial receptivity and improving successful implantation rates in in vitro fertilization (IVF) treatments. Ren et al. have investigated dynamic changes in various cell components within endometrium during the transition from normal to endometrial cancer to provide valuable insights into cellular origins of endometrial cancer and identified specific subpopulations associated with the tumorigenic process using scRNA-Seq [60]. Through analysis, they discovered that endometrial cancer originated from epithelial cells rather than stromal cells. More specifically, they identified unciliated glandular epithelium as cellular source of endometrial cancer [60]. Furthermore, they identified a distinctive subpopulation of cells that might play a crucial role in tumor development. These cells were characterized by the expression of LCN2, SAA1, and SAA2 genes. These findings contribute to the broader understanding of endometrial cancer pathogenesis with potential to inform the development of targeted therapies. Kirkwood et al. have employed scRNA-Seq to investigate the process of endometrial repair in a mouse model. They examined transcriptomic profiles of three different transgenic mouse models and identified a previously unknown subpopulation of PDGFRb^+^ stromal-like stem cells that exhibited distinct transcriptomic changes specifically in response to endometrial dysfunction or damage [14]. They also demonstrated that the plasticity and versatility of stromal fibroblasts could contribute to the restoration of the endometrium's structural integrity by undergoing a mesenchyme to epithelial transformation. Jiang et al. have explored dynamic changes occurring in mouse endometrial tissues during the post-implantation stage. They made significant discoveries regarding the involvement of different subtypes of endometrial stromal-like cells that played crucial roles in extracellular remodeling during implantation [61]. Furthermore, their study shed light on communication and interactions between endometrial stromal cells, epithelial cells, and immune cells during the implantation process. They also revealed that these stromal cells engaged in communication with epithelial cells and immune cells through nectin and ICAM signaling pathways during implantation.

### Assay for transposase-accessible chromatin using sequencing (ATAC-seq)

**Fig. 4:**
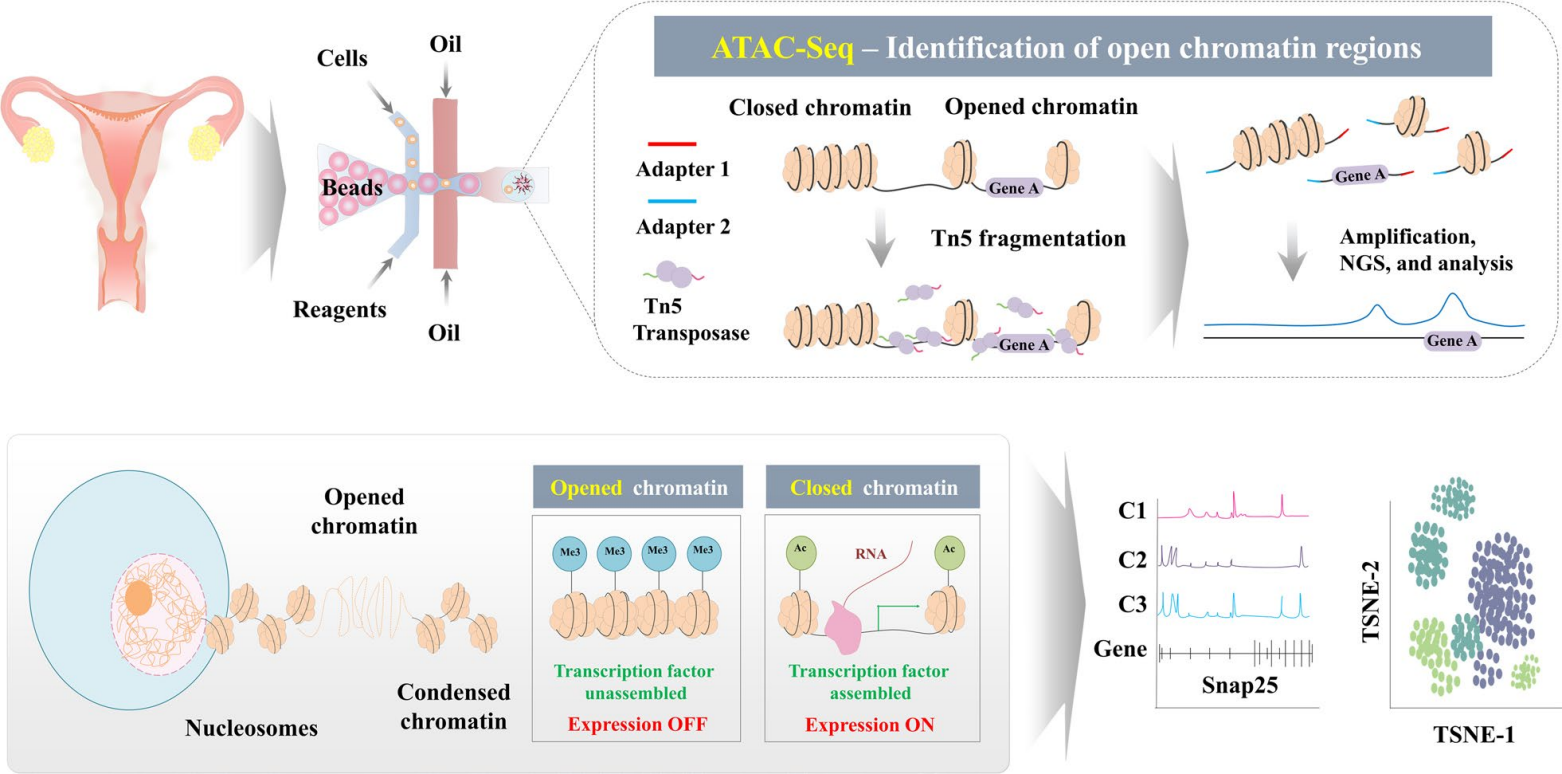
Illustrative depiction of the methodology behind single-cell ATAC sequencing (scATAC-Seq) and the intricate mechanisms impacting chromatin accessibility. Chromatin accessibility, a pivotal indicator of a cell's regulatory landscape, is profoundly shaped by a convergence of molecular events. At the DNA level, the methylation and acetylation of specific sites intricately modulate the affinity of diverse proteins, including transcription factors and enzymes involved in histone modification. The cumulative effect of these molecular interactions contributes to the selective silencing of particular genomic regions, orchestrating the cell's gene expression program. The scATAC-Seq employs a hyperactive variant of the Tn5 transposase to elucidate accessible chromatin regions. Consequently, during scATAC-Seq, the genome is treated with this modified Tn5 transposase to identify open chromatin regions, revealing the dynamic accessibility of various genomic loci. The figures presented in this article were crafted by our group

In eukaryotic cells, DNA is packaged and organized by histones to form chromatin, a highly dynamic structure that undergoes reversible chemical modifications. These modifications primarily include DNA methylation and histone post-translational modifications. They play crucial roles in various biological processes, including gene regulation, genomic imprinting, and chromatin stability [62]. Gene expression is regulated by accessibility of chromatin, which is achieved through modulating interactions between their target DNAs and transcription factors. Chromatin modifications play a crucial role in determining the packing level of chromatin, thereby influencing its accessibility. These modifications include DNA methylation, histone acetylation, methylation, phosphorylation, and many others [63]. By altering the chromatin structure, these modifications can regulate the accessibility of transcriptional factors to their target DNA sequences, ultimately affecting gene expression patterns [64]. Euchromatin characterized by open and accessible regions is particularly associated with the pluripotency of embryonic stem cells. In contrast, heterochromatin regions tend to increase during cellular differentiation processes, leading to a more compact and repressed chromatin state [65, 66]. Understanding the dynamic nature of epigenetic modifications and their impact on gene expression is crucial for unraveling complexities of developmental processes, cellular differentiation, and disease etiology [67]. Advancements in epigenomic profiling technologies such as DNA methylation sequencing and chromatin immunoprecipitation sequencing (ChIP-Seq) have provided valuable tools to investigate the epigenetic landscape and its functional implications. In addition to these conventional epigenomic profiling platforms, assay for transposase-accessible chromatin using sequencing (ATAC-seq) has emerged as a transformative technique for characterizing the gene regulatory landscape and quantifying chromatin accessibility at a single-cell level. Its ability to quantify chromatin accessibility at high resolution, its applicability to heterogeneous samples, and its compatibility with other single-cell techniques make it an invaluable tool for deciphering complex mechanisms governing gene regulation in health and diseases [38] (Fig. 4).

Decidualization, a crucial process in mammalian pregnancy, involves transformation of undifferentiated endometrial stem cells into specialized decidual cells. This transformation occurs when the implanting embryo breaches the luminal endometrial epithelium. Decidual cells play a vital role in establishing a protective and nutritive environment around the developing embryo, facilitating controlled trophoblast invasion and ensuring maternal immune tolerance of the antigenically distinct fetus [68]. Despite the significance of decidualization, the precise mechanisms governing the dynamic change of undifferentiated endometrial stem cells to decidual cells in the chromatin landscape during this process remain largely uncharacterized. In this context, Vrljicak et al. have investigated chromatin accessibility profiles in undifferentiated endometrial stem cells and upon decidualization using ATAC-Seq. They revealed a notable reduction in chromatin accessibility during the process of decidualization [69]. This reduced accessibility was specifically associated with loss of binding motifs for certain transcription factors (TFs) known to be repressed upon decidualization. Runt-related transcription factors 1 and 2 (RUNX1 and RUNX2), ETS Proto-Oncogene 1 (ETS1), and SRY-box 12 (SOX12) are among TFs with diminished binding motifs [69]. Their study provided valuable insights into chromatin-level changes underlying the process of decidualization in human endometrial stem cells.

The combination of scRNA-Seq and scATAC-Seq represents a powerful approach that allows for high-resolution investigation of complex epigenetic events in tumor biology. This integrated approach not only enables identification and classification of distinct cell types within a tumor, but also provides insights into underlying mechanisms and pathways driving tumorigenesis beyond traditional taxonomic classifications. Therefore, Regner et al. were able to gain insights into the intratumoral heterogeneity and its impact on gene expression regulation by generating matched transcriptome and chromatin accessibility profiles at the single-cell level using scRNA-Seq and scATAC-Seq combination [10]. They observed substantial variations in chromatin accessibility among malignant cells derived from the same patients. This variation in chromatin accessibility was found to be directly linked to transcriptional output, indicating that changes in chromatin structure might play a crucial role in driving gene expression patterns within tumors.

### Spatial transcriptomics

**Fig. 5:**
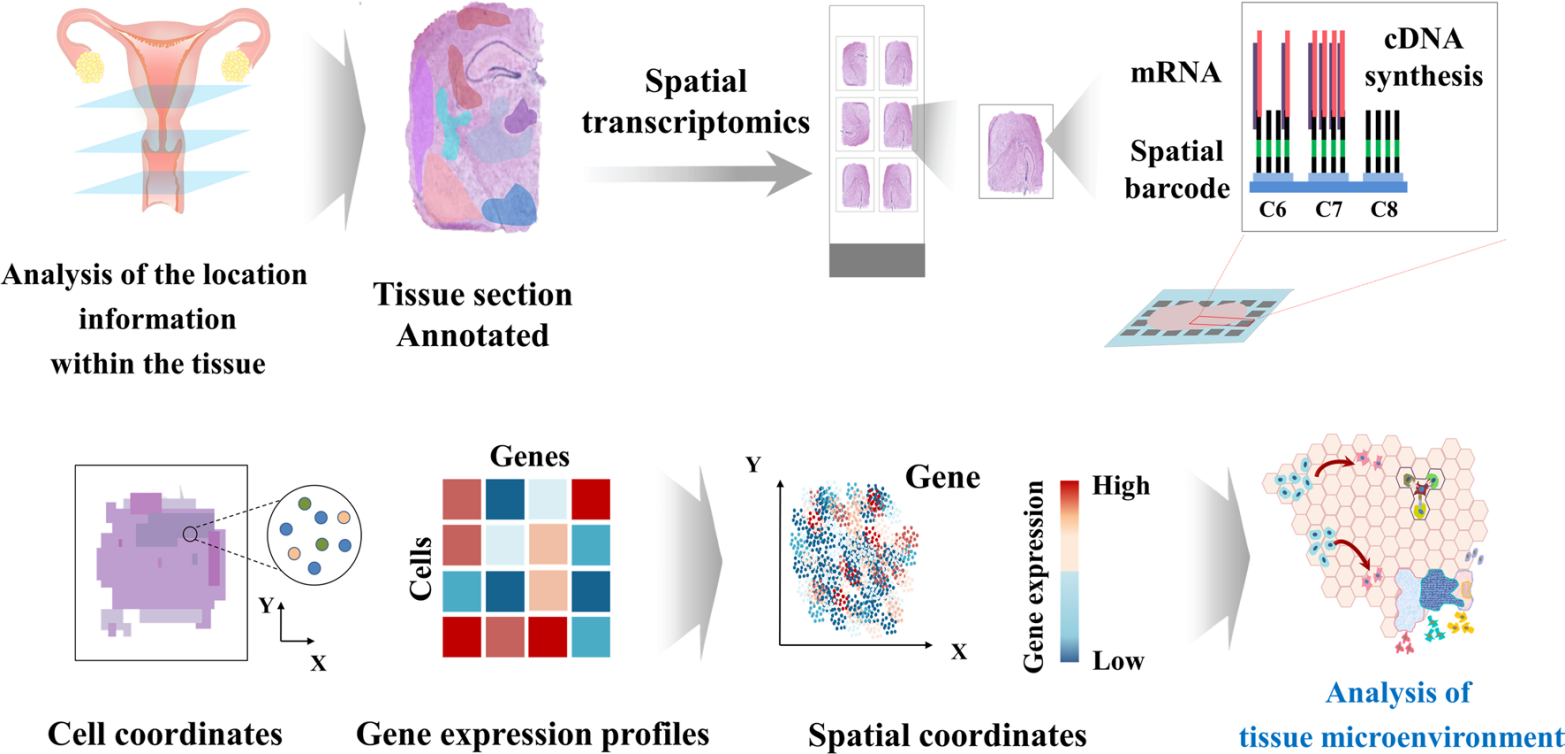
A comprehensive depiction of spatial transcriptomic analysis. Spatial Transcriptomics represents a pioneering approach that facilitates a meticulous exploration of gene expression patterns within the context of tissue sections. By capturing high-resolution gene expression data while preserving the intricate tissue architecture, spatial transcriptomic datasets not only provide precise gene expression measurements but also impart a profound understanding of gene activity within its native tissue microenvironment. The figures presented in this article were crafted by our group

Recent advancements in scRNA-Seq technologies have enabled exploration of single-cell transcriptome in various contexts, including human endometrial tissue and mouse uterus throughout different menstrual cycle and pre-/post-implantation [42, 51, 70, 71]. While scRNA-Seq provides valuable insights into cellular heterogeneity and gene expression profiles at the single-cell level, it lacks spatial information, which is lost during single-cell isolation process [72]. This limitation hinders comprehensive understanding of cellular interactions within the tissue. To overcome this challenge, spatial transcriptomic technologies, which allow for spatial assessment of gene expression patterns at a single-cell levels within intact tissue sections, have garnered significant attention [73]. By preserving the spatial context of cells, spatial transcriptomics not only provides insights into the localization and heterogeneity of cell populations within the endometrium, but also reveals how neighboring cells influence the behavior and function of individual cells [74, 75] (Fig. 5).

Garcia-Alonso et al. have performed an in-depth investigation into cellular states and spatial organization of human endometrial cells during different phases of the menstrual cycle in women of reproductive age integrating scRNA-Seq and spatial transcriptomic profiling [11]. They identified specific spatial coordinates associated with distinct subsets of cells expressing transcription factor SOX9. They observed that noncycling SOX9^+^/LGR5^+^ cells were predominantly enriched in the surface epithelium of the endometrium. On the other hand, noncycling SOX9^+^/LGR5^−^ cells were primarily located within basal glands. Additionally, their study revealed that cycling SOX9^+^ cells were predominantly mapped to glands within the growing superficial layer of the endometrium. These findings have implications for understanding dynamic changes occurring in the endometrium throughout the menstrual cycle and provide a foundation for further investigations into roles of these specific cell subsets in endometrial function, regeneration, and establishment of receptive conditions for embryo implantation. Li et al. have employed a combination of spatial transcriptomics and scRNA-Seq analyses to investigate local gene expression patterns at the site of implantation on pregnancy [76]. This approach allowed them to gain a comprehensive understanding of gene expression patterns and cellular compositions within the microenvironment at this critical stage of pregnancy. Spatial transcriptomic analysis enabled the characterization of 11 distinct domains, each characterized by unique gene signatures [76]. Yu et al. have also utilized a combination of spatial transcriptomics data scRNA-Seq datasets to investigate cellular compositions and molecular interactions within endometrial cancer tissue slices [77]. They observed that two subclusters of epithelial cells, namely blood endothelial cells and lymphatic endothelial cells, exhibited a more malignant phenotype. Their malignant phenotype might be conferred through activation of the MK pathway by MDL-NCL signal cascades [77]. This signaling mechanism potentially plays a role in promoting malignancy in endothelial cells associated with endometrial carcinoma. Notably, NCL was found to be associated with suppressed immune activity, indicating a potential mechanism through which endometrial carcinoma cells could inhibit immune cells within the tumor microenvironment. The integration of spatial transcriptomics and scRNA-Seq data provides valuable insights into molecular interactions and cellular heterogeneity within endometrial cancer, contributing to our understanding of tumor progression and potential therapeutic targets.

## Conclusions

Single-cell analysis has emerged as a powerful tool for investigating properties of endometrial stem cells. For instance, scRNA-Seq has unveiled dynamic changes in the functionalis layer during the menstrual cycle, providing potential biomarkers for endometrial receptivity and aiding in IVF treatments. In the context of endometrial cancer, scRNA-Seq identified specific cellular origins and subpopulations associated with tumorigenesis, informing potential targeted therapies. Additionally, scRNA-Seq has been instrumental in studying endometrial repair, post-implantation dynamics, and communication between stromal cells, epithelial cells, and immune cells. Combining scRNA-Seq and scATAC-Seq offers a powerful approach to investigating epigenetic events in tumor biology, uncovering intratumoral heterogeneity and its impact on gene expression regulation. Integrating scRNA-Seq and spatial transcriptomics has identified specific cellular states and spatial coordinates in the endometrium throughout the menstrual cycle. This approach has also been applied to study implantation sites during pregnancy and explore molecular interactions and cellular heterogeneity in endometrial cancer, contributing to our understanding of tumor progression and potential therapeutic targets.

However, there are still some limitations that need to be addressed to fully harness the potential of single-cell analysis in endometrial stem cell research. One major limitation is the lack of standardization in sample preparation and data analysis. Single-cell analysis involves isolation of individual cells, which can be challenging due to technical variations and heterogeneity of endometrial tissues that limit the accuracy and reproducibility of downstream analyses. To overcome these challenges, several strategies have been developed for cell isolation, including fluorescence-activated cell sorting (FACS) and magnetic-activated cell sorting (MACS). FACS and MACS relying on labeling of cells with fluorescent or magnetic markers, respectively, are widely used methods for cell isolation. These methods enable the selection and isolation of specific cell populations based on their surface markers or other molecular features. In addition, the use of multiple complementary cell isolation methods such as FACS and MACS can help validate and cross-reference results from single-cell analysis studies. Furthermore, the integration of cell isolation with other single-cell analysis techniques such as spatial transcriptomics and multi-omics approaches can provide a more comprehensive and detailed understanding of molecular and functional properties of endometrial stem cells.

Other limitations of single-cell analysis include its high cost and complexity. The equipment and reagents required for single-cell analysis can be expensive and the data generated from single-cell analysis can be complex and difficult to interpret. One major cost driver in single-cell analysis is the cost of sequencing, which can be a significant expense for studies that require large-scale sequencing of individual cells. To overcome these cost limitations, several strategies have been developed to reduce the cost of single-cell analysis. One approach is to use targeted sequencing methods such as single-cell targeted sequencing (scTSS) to enable sequencing of a focused set of genes or genomic regions at a lower cost than whole-genome or whole-transcriptome sequencing. Another approach is to use pooling strategies such as cell hashing or split-pool barcoding to enable sequencing of multiple cells in a single sequencing reaction, thereby reducing the cost per cell. In addition, the use of efficient data processing and analysis pipelines, such as those based on machine learning or deep learning algorithms, can help reduce computational resources required for data analysis.

Despite these limitations, various single-cell analysis platforms have been used to investigate endometrial stem cells. One of the main advantages of single-cell analysis is that it can reveal cell-to-cell variations that might be masked by bulk analysis. By analyzing individual cells, researchers can identify rare cell types or subpopulations that might be missed by bulk analysis. They can also identify cell-to-cell variations in gene expression or other cellular features that might be important for understanding stem cell function. In addition, by analyzing gene expression patterns of individual cells over time, researchers can identify key signaling pathways and regulatory factors that drive stem cell differentiation. They can also investigate the role of environmental cues in modulating stem cell fate. In the future, there is a need for more comprehensive and integrated single-cell analysis approaches that can simultaneously measure multiple aspects of endometrial stem cells, such as their gene expression, epigenetic modifications, protein expression, and functional properties. Additionally, the development of new technologies for single-cell analysis, such as spatial transcriptomics and multi-omics approaches, will be important for advancing our understanding of endometrial stem cell biology and their roles in reproductive health and disease.

## Data Availability

Not applicable.

## Abbreviations

ChIP-Seq: Chromatin immunoprecipitation sequencing
CXCL14: C-X-C motif chemokine ligand 14
DIO2: Iodothyronine deiodinase 2
ECM: Extracellular matrix
ETS1: ETS proto-oncogene 1
FACS: Fluorescence-activated cell sorting
GPX3: Glutathione peroxidase 3
ICAM: Intercellular adhesion molecule
IVF: In vitro fertilization
LCN2: Lipocalin-2
LGR5: Leucine rich repeat containing G protein-coupled receptor 5
MACS: Magnetic-activated cell sorting
MCAM: Melanoma cell adhesion molecule
PAEP: Progestagen associated endometrial protein
PDFGRB: Platelet derived growth factor receptor Beta
RPL: Recurrent pregnancy loss
RUNX1: RUNX family transcription factor 1
SAA1: Serum amyloid A1
scATAC-Seq: Single-cell ATAC sequencing
scRNA-Seq: Single-cell RNA sequencing
scTSS: Single-cell targeted sequencing
SCARA5: Scavenger receptor class A member 5
SOX9: SRY-box transcription factor 9
TFs: Transcription factors
WOI: Window of implantation
